# The immune-epithelial interface in eosinophilic esophagitis: a conversation

**DOI:** 10.3389/falgy.2023.1270581

**Published:** 2023-10-03

**Authors:** David A. Hill, Amanda B. Muir

**Affiliations:** ^1^ Division of Allergy and Immunology, Children's Hospital of Philadelphia, Philadelphia, PA, United States; ^2^ Department of Pediatrics and Institute for Immunology, Perelman School of Medicine at the University of Pennsylvania, Philadelphia, PA, United States; ^3^ Division of Gastroenterology, Children's Hospital of Philadelphia, Philadelphia, PA, United States; ^4^ Department of Pediatrics, Perelman School of Medicine at the University of Pennsylvania, Philadelphia, PA, United States

**Keywords:** eosinophilic esophagitis, esophageal epithelial cells, epithelium, food allergy, type 2 inflammation

## Two sides of the same coin

**“**Eosinophilic esophagitis (EoE) is an immune-mediated disease of the esophagus”… is how hundreds of articles, reviews, and clinical guidelines have introduced EoE over the past several decades—and with good reason! There is unequivocal evidence of the immune system's role in EoE well beyond its *sine qua non* of esophageal eosinophilia (1). Clinically, murine and human studies support that a large proportion of EoE is the result of transepithelial antigen exposure as mice will develop EoE-like inflammation after epidermal sensitization and children with atopic dermatitis are at increased risk of EoE development (2–5). There is also robust molecular evidence of immune involvement in EoE including transcriptomic (6–8) and mechanistic (9, 10) studies of type 2 (T2) inflammatory pathways such as those mediated by IL-5 (11) and IL-13 (12). Most recently, in-depth interrogations of patient T cells have established the role of the adaptive immune system in EoE (13–15). Taken together, this body of work has formed the basis of our understanding of EoE immunopathology and led to the utilization of steroids, T2-targeting biologics, and other immune-modulatory medications as the backbone of EoE therapy (16).

However, key leaders in the field have focused attention on EoE as a disease of the epithelium (17). This perspective is justified by recent data that has identified epithelial cells as critical to EoE pathogenesis. For example, many of the most high-risk disease loci for EoE encode epithelial proteins including calpain 14, thymic stromal lymphopoietin (TSLP), Desmoglein 1, Filaggrin, and STAT6 (12, 17–20—all) of which have been shown to be relevant to epithelial barrier integrity and/or T2 inflammation (2, 21). Furthermore, genetic and functional data establish a primary role for impaired epithelial barrier function in disease susceptibility and pathoetiology (21–24). Additionally, the EoE transcriptome (a set of genes dysregulated in the esophagi of patients with EoE) is enriched in genes that encode for proteins involved in esophageal epithelial cell differentiation (8, 25). Taken together, these studies suggest that the epithelium is more than just a passive respondent to the inflammation of EoE, but rather an active participant.

## The Mucosa as a barrier

Integrity of the mucosal barrier throughout the gastrointestinal tract is critical as it provides protection against invading microbes and exposure to harmless food proteins. In the esophagus, barrier function is maintained by a stratified epithelium in which differentiation from basal to squamous cells is exquisitely regulated. The epithelium is made up of basal cells (which are positioned atop the lamina propria), a proliferative layer of transit amplifying cells, and more apical layers of increasingly differentiated cells that become anucleate and eventually slough off into the lumen. Molecular evidence in the form of single cell analysis of the active EoE esophageal epithelium has demonstrated a halted differentiation process that persists in remission despite decreased inflammation (8). Further, clinical studies demonstrate that there are disruptions in normal differentiation in EoE with basal cell hyperplasia (BCH), as well as decreased expression of tight junctions, leading to dilated intercellular spaces (26). These changes are attenuated as the inflammation decreases in the setting of disease remission, however, differentiation does not fully normalize (8, 27). Even in the setting of remission, there remains persistent basal cell hyperplasia in 28% of patients with EoE, and patients with persistent basal cell hyperplasia have increased symptomatology compared to those who regain normal differentiation (8, 27). Together, these observations demonstrate that there are primary defects in the EoE epithelium that are independent of the degree of inflammation.

## Epithelial cells as immune sentinels

Like epithelial cells elsewhere in the gastrointestinal tract (28), esophageal epithelial cells (EECs) themselves can act as detectors of inflammatory stimuli and directors of inflammatory responses. EECs are ideally located for immunological surveillance as they have the potential to sample food components, commensal and pathogenic microorganisms, and toxins from luminal contents. EECs express several innate pathogen recognition receptors (PRRs) including Toll-like receptors (TLR) (29, 30), NOD-like receptors (31, 32), and G-protein-coupled receptors (33). We know that these pathways are both active and relevant to EEC biology as stimulation of EECs with TLR ligands augments esophageal barrier integrity (30).

There is also emerging evidence that under pathological conditions, EECs (like other gastrointestinal epithelial cells) can act as non-professional antigen presenting cells and modulate adaptive immune responses. For example, EECs from patients with active EoE express major histocompatibility complex (MHC) class II, CD80, and CD86 (34). Notably, both elevated interferon-*γ* (IFN*γ*) levels and elevated IFN*γ* response gene signatures have been detected in active EoE (34, 35). This is relevant as IFN*γ* is known to induce MHCII expression by non-professional APCs in other settings (36). Indeed, IFN*γ* stimulation of the human esophageal epithelial line HET-1A increases expression of MHCII, as well as the processing and presentation of ovalbumin and causes T helper cell activation (34).

Finally, EECs can both sense and release cytokines and chemokines to augment their own and the immune system's response to food antigens. For example, loss of tonic regulatory signals, such as TGF*β*, can lead to hyperproliferation, failure of differentiation, and overexpression of innate proinflammatory mediators by EECs (37). Further, IL-13 signaling on EECs leads to induction of an EoE-like transcriptional program (25), increased epithelial protease activity, and impaired barrier function (38, 39). EECs are also a critical source of early, innate inflammatory cytokines such as TSLP and IL-33 (2, 40–42), which direct esophageal inflammatory responses (24, 43). Together, these data highlight the central role for EECs, and the epithelium in general, as central modifiers of the mucosal immune response.

## The conversation

**Figure 1:**
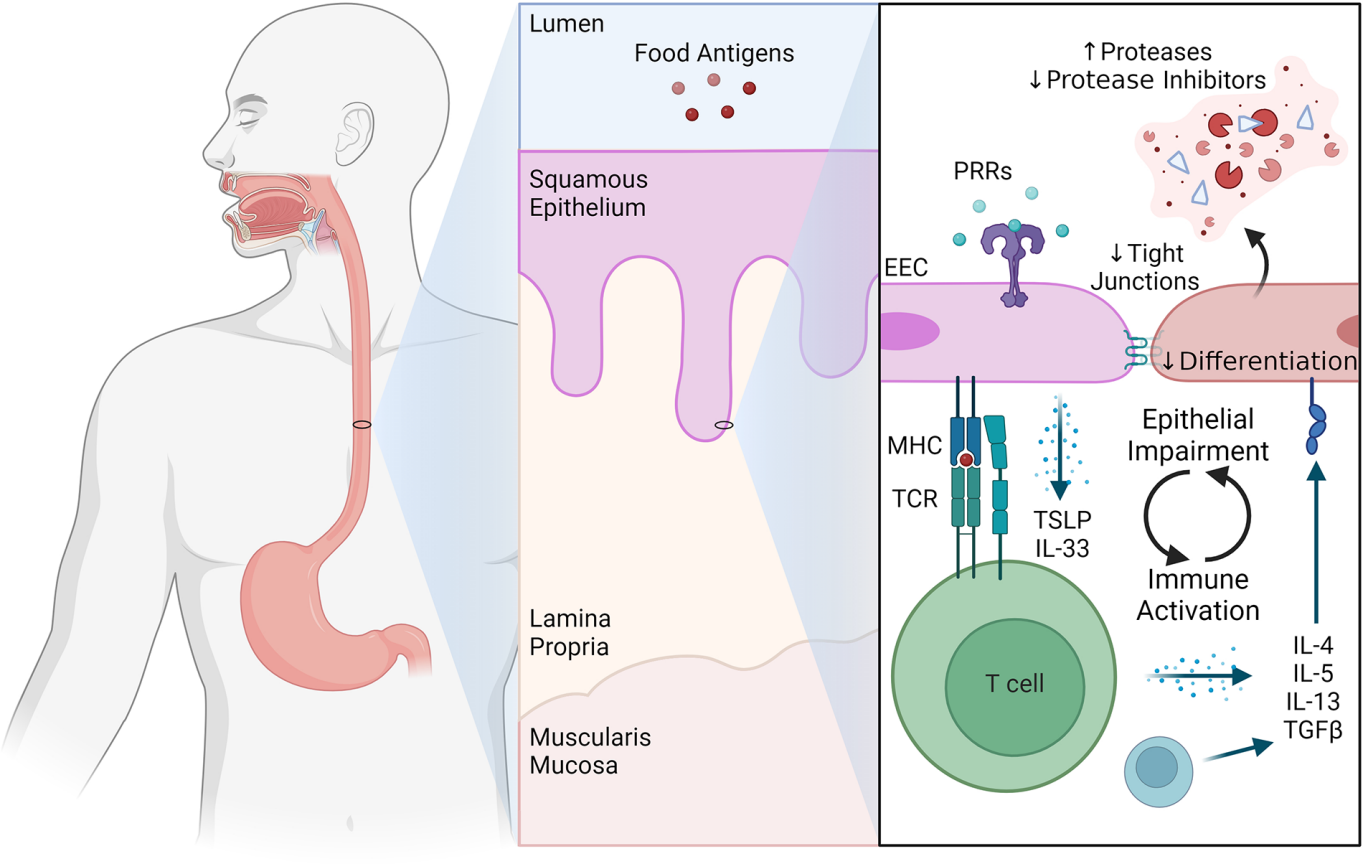
The immune-epithelial interface in eosinophilic esophagitis.

Understandably, immunologists and epithelial cell biologists have historically focused on their respective areas of expertise when arguing the relative importance of the immune system or the epithelium to EoE pathogenesis. However, the truth is likely somewhere in the middle: that EoE represents the culmination of a complex and dynamic conversation between epithelial cells and the immune system (Figure 1). As an extension, investigations of the immune-epithelial interface in EoE will provide new discoveries that can be exploited therapeutically to enhance beneficial and abrogate pathogenic communication between immune and epithelial cells.

Future research efforts in this space would be best served by collaborative teams of immunologists and epithelial cell biologists that can complement each other to drive new and innovative science at the immune-epithelial interface. Specific areas of focus could include: (1) identification of novel molecules and pathways that mediate the bidirectional crosstalk between epithelial and immune cells; (2) more studies of the role that EECs play in antigen sampling, presentation, and modulation of adaptive immune responses; and (3) longitudinal studies of the immune-epithelial interface to understand how it changes during the transition from acute to chronic disease. In doing so, researchers should consider limitations and biases that can accompany investigations of the esophageal epithelium including that biopsies are taken at random, are limited to the epithelium providing inadequate sampling of lamina propria and muscularis (44), and as a result mostly contain epithelial cells and less fibroblast or nerves which may skew our understanding of the disease etiology. Hopefully, functional evaluations of esophageal distensibility and motility (e.g., functional lumen imaging probe and manometry) will improve our understanding of esophageal dysfunction below the epithelial surface (45). These research efforts will be accelerated by requests for collaborative research proposals by the NIH focused on the immune-epithelial interface of EoE. Ultimately, this line of research has the potential to introduce a new class of EoE-specific therapeutics to the field that can complement immune or epithelial-targeted medications to treat refractory endotypes and improve clinical outcomes.
